# Analysis of the Costs Associated With the Elective Evaluation of Patients Labelled as Allergic to Beta-Lactams or Nonsteroidal Antiinflamatory Agents

**DOI:** 10.3389/fphar.2020.584633

**Published:** 2020-11-30

**Authors:** Miriam Sobrino-García, Esther M. Moreno, Francisco J. Muñoz-Bellido, Maria T. Gracia-Bara, Elena Laffond, Inmaculada Doña, Cristina Martín, Eva M. Macías, Sonia de Arriba, Valle Campanón, Alicia Gallardo, Ignacio Dávila

**Affiliations:** ^1^ Allergy Service, University Hospital of Salamanca, Salamanca, Spain; ^2^ Institute for Biomedical Research of Salamanca (IBSAL), Salamanca, Spain; ^3^ Department of Biomedical and Diagnostic Sciences, Faculty of Medicine, University of Salamanca, Salamanca, Spain; ^4^ Asthma, Allergic and Adverse Reactions (ARADyAL), Network for Cooperative Research in Health of Instituto de Salud Carlos III, Salamanca University Hospital, Salamanca, Spain; ^5^ Allergy Service, University Hospital of Malaga, Malaga, Spain; ^6^ Biomedical Research Institute of Malaga (IBIMA), Malaga, Spain

**Keywords:** beta-lactam, cost, delabelling, drug hypersensitivity, non-steroidal anti-inflammatory drug, penicillin, drug allergy

## Abstract

**Introduction:** Being labelled as allergic to different drugs results in patients receiving other treatments, which are more toxic, less effective and more expensive. We aimed to analyze different studies of the costs of drug hypersensitivity assessment.

**Methods:** A bibliographic search on studies regarding this issue was performed, including the available scientific evidence up to June 2020. We searched three databases with terms related to costs and allergy testing in drug hypersensitivity reactions.

**Results:** Our search revealed 1,430 publications, of which 20 met the inclusion criteria. In the manuscript, prospective studies evaluating the costs of the evaluation of patients with suspected allergy to beta-lactams or non-steroidal anti-inflammatory drugs are analyzed. Also, comment is made on the costs associated with incorrect labeling as non-steroidal anti-inflammatory drug or penicillin hypersensitivity.

**Conclusions:** Taking all costs into account, the study of drug hypersensitivity is not expensive, particularly considering the economic and clinical consequences of labeling a patient with hypersensitivity to drugs.

## Introduction

Drug allergy can affect 7–10% of the general population and constitutes a Public Health issue (Park et al., 2011; Macy and Ngor, 2013; Sagar and Katelaris, 2013). Nevertheless, most patients that claim to have drug hypersensitivity are not really allergic after an allergological study (Park et al., 2011; Macy and Ngor, 2013; Sagar and Katelaris, 2013).

Beta-lactams are one of the drugs most usually implicated in adverse immunological reactions (Bedolla-Barajas et al., 2018). An unverified penicillin allergy results in patients receiving broader-spectrum antibiotics that are frequently less clinically and economically effective. In addition, the unnecessary use of alternative antibiotics leads to more adverse reactions, treatment failures, and healthcare infections (MacLaughlin et al., 2000; Sade et al., 2003; Shehab et al., 2008; Picard et al., 2013; Macy and Contreras, 2014; McDanel et al., 2015; Barlam et al., 2016).

Regarding children, about 10% of parents state that their children are allergic to drugs, especially to beta-lactams, probably related to high prescription rates (Atanaskovic-Markovic et al., 2019; Calamelli et al., 2019; Roduit, 2019). Nevertheless, only a small proportion of them are true drug allergic reactions (Macy and Ngor, 2013). In this sense, different studies concluded that fewer than 10% of patients claiming to be allergic really are, so most children are mislabeled as drug allergic (Seitz et al., 2011; Abrams et al., 2016; Mill et al., 2016).

Non-steroidal anti-inflammatory drugs (NSAIDs) are one of the most frequent drugs causing hypersensitivity reactions with a prevalence of 1–3%.This is higher in patients with chronic rhinosinusitis, nasal polyposis, asthma, or chronic urticaria, rising to 30% (Wöhrl, 2018). The importance of its evaluation is because the necessity of NSAIDs for analgesic/anti-inflammatory or antiplatelet therapy (Modena et al., 2017).

Nevertheless, the costs associated with the evaluation of patients claiming to be allergic to drugs should be considered. In this review, the costs of evaluating drug hypersensitivity in beta-lactam antibiotics and NSAIDs are analyzed.

Thus, the review aims were to analyze different studies of the costs of drug hypersensitivity assessment, particularly prospective studies of the evaluation of beta-lactam allergy in adults and children patients, NSAID hypersensitivity in adults, and other studies revealing the clinic and economic consequences and the importance of delabelling.

## Methods

### Literature Search

A bibliographic search on studies regarding this issue was performed including the available scientific evidence up to June 2020. The primary sources for the search included PubMed, SCOPUS, and EMBASE.

The search terms for PubMed included (“costs and cost analysis” [MeSH Terms] OR “cost-benefit analysis” [MeSH Terms]) AND “allergy testing” [Other Term]) OR “allergy tests” [Other Term]) OR “allergy evaluation” [Other Term]) OR “delabelling” [Other Term]) AND “penicillin” [Other Term]) OR “beta-lactam” [Other Term]) OR “nsaid” [Other Term]. Similar terms and methods were used for the other databases.

### Inclusion and Exclusion Criteria

Only original articles or systematic reviews were selected.
Non-systematic reviews, comments, and other types of articles were not selected.
Only articles in English were considered.
Only articles explicitly dealing with hypersensitivity reactions were included.
No age restriction was considered.
At least two blinded researchers independently reviewed titles and abstracts from the initial search, and eligibility criteria determined their inclusion or exclusion.

Prospective studies about the costs of evaluating patients labeled as allergic to different drug such as beta-lactam in adults (Βlumenthal et al., 2018; Sobrino-García et al., 2019) and children (Sobrino et al., 2020) or NSAIDs (Sobrino-García et al., 2020) drive this field forward prioritized.

## Results

**FIGURE 1:**
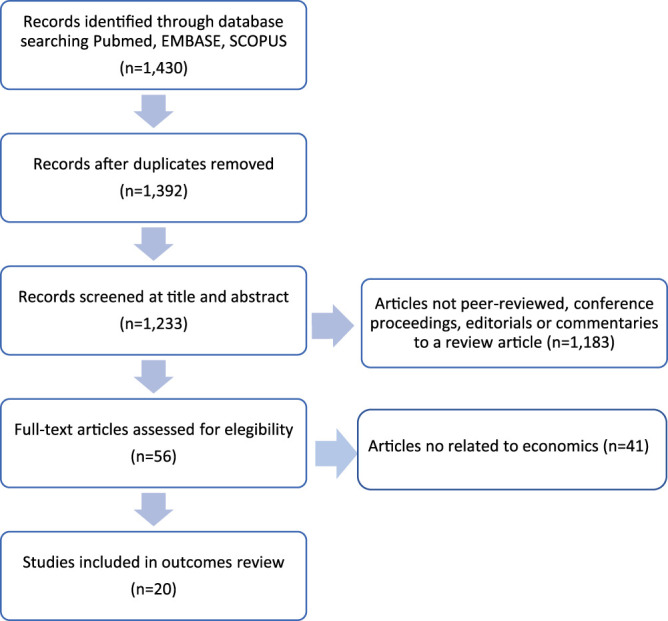
Flow diagram for search methods.

Firstly, database searches showed 1,430 results. After removing duplicates and articles without abstracts, 1,233 abstracts remained for screening. Articles not peer-reviewed, conference proceedings, editorials or commentaries to review articles were excluded after abstracts were evaluated. Other articles were excluded because they did not explicitly analyze costs in drug allergy. After applying inclusion and exclusion criteria, 20 articles were included in the review (Figure 1).

### Assessment of Costs

We only found three prospective articles evaluating patients with suspected beta-lactam allergy, two of them performed in adults, and one in children. There was also a prospective study evaluating the costs of the evaluation of hypersensitivity to NSAIDs in adults. Direct and indirect costs as explained by Soto-Álvarez (2020) were systematically recorded in only a few studies(Βlumenthal et al., 2018; Sobrino et al. 2020; Sobrino-García et al., 2019; Sobrino-García et al., 2020). Direct health costs were calculated by Βlumenthal et al. (2018), who analyzed personnel costs, consumables and space costs. Moreover, other studies (Sobrino et al., 2020; Sobrino-García et al., 2019; Sobrino-García et al., 2020) considered the number of consultations and all the diagnostic tests performed. Direct non-health costs (Sobrino et al., 2020; Sobrino-García et al., 2019; Sobrino-García et al., 2020) were calculated taking into account the number of consultations and the distance from their homes to the Allergy service. Finally, indirect costs (Sobrino et al., 2020; Sobrino-García et al., 2019; Sobrino-García et al., 2020) were calculated considering the absenteeism of patients.

In the US, Βlumenthal et al. (2018) prospectively calculated the cost of evaluating beta-lactam allergy in outpatients and found a cost of $220 (€209.37) for a case that included penicillin skin tests and a drug provocation test (DPT) in one step with amoxicillin. In cases where more resources were used, assessment of penicillin allergy costs were around $540 (€482.45). In Europe (Spain, Sobrino-García et al., 2019) performed a prospective, one-year-long study in 296 patients (46 positives), obtaining an average cost of €187.49, with a maximum cost of €789.96.

In children, the only prospective study (Sobrino et al. 2020), performed in Europe and one year lasting, evaluated 40 children with suspected beta-lactam allergy (with only three children finally diagnosed with beta-lactam allergy). The mean cost was €275.27 with a maximum cost of €746.78.

Concerning NSAIDs the only prospective elective study (Sobrino-García et al., 2020) included 233 patients (43 positives at the end of the study), with an average cost of €185.30 (maximum €1,055.96).

## Discussion

Prospective studies concerning the costs of the elective evaluation of drug allergy are scarce. Thus, two studies evaluated the costs of the elective evaluation of adult patients labeled with beta-lactam allergy (Βlumenthal et al., 2018; Sobrino-García et al., 2019)Another study evaluated such costs in children (Sobrino et al. 2020) and yet another analyzed the costs of the elective evaluation of adults labeled as hypersensitive to NSAIDs (Sobrino-García et al., 2020). One of them was performed in the United States (Βlumenthal et al., 2018) and the other three in Europe (Sobrino et al., 2020; Sobrino-García et al., 2019; Sobrino-García et al., 2020).

### Beta-Lactam Studies in Adults

European studies (Sobrino et al., 2020; Sobrino-García et al., 2019; Sobrino-García et al., 2020) were performed following the ENDA/EAACI protocols (Torres et al., 2003; Kowalski et al., 2011; Moreno et al., 2016). According theses protocols in beta-lactam allergy evaluation included the anamnesis followed by STs. When negative, patients underwent DPT.

In one of the beta-lactam allergy studies (Sobrino-García et al., 2019) performed, additional visits for challenging with alternative beta-lactams were done. All consultations were prospectively collected. In the prospective beta-lactam study performed in the United States, Βlumenthal et al. (2018) (United States), who studied 30 outpatients with suspected beta-lactam allergy, the mean cost was $220 (€209.37) for the base case (skin tests with beta-lactam and DPT with amoxicillin). In cases where more resources were used and testing was expanded, assessment of penicillin allergy would only cost about $540 (€482.45).

The results of both studies are not entirely comparable for several reasons (World Bank, 2019): i) per capita income of the United States and Spain are very different (€53,341 and €25,900, respectively, in 2018), ii) there are apparent differences between both countries regarding their National Health System (mainly private in the United States and predominantly public in Spain); iii) the differences between protocols, and iv) the exchange rate between the euro and the dollar, which means that amounts obtained require a conversion to be able to compare them (1 EUR = 1.1250 USD). Thus, another study (Sousa-Pinto et al., 2020a) designed an online questionnaire to study the practice and cost perceptions of diagnostic tests used in beta-lactam allergy evaluation. They concluded that there is a great deal of diversity in performing beta-lactam allergy studies and reported cost estimates, with median values ranging from €50 for SPT to €190 for DPT and providing information on the need for context-based cost assessments and when these studies can be economically effective. Sobrino-García et al. studied different factors that influenced the costs of their studies (Sobrino et al., 2020; Sobrino-García et al., 2019; Sobrino-García et al., 2020). In the study of beta-lactams in adults (Sobrino-García et al., 2019), the costs in patients with immediate reactions (€152.64 ± 106.73) and delayed reactions (€220.48 ± 171.79), were significantly different (*p* < 0.001). This fact was mainly related to the mean number of visits (1.95 ± 0.90 and 2.76 ± 1.30, respectively, *p* < 0.001). For patients who worked and did not work for hire, wages were also different (*p* < 0.001), with a mean loss of income of €364.12 ± 156.38 and €121.28 ± 68.18, respectively.

Moreover, Sousa-Pinto et al. (2020b) concluded in a recent study that penicillin allergy evaluation was cost-saving in twenty-four base case decision models. For models evaluating the performance of skin tests and DPT, allergy testing involved an average savings of $657 for inpatients (USA: $1,444; Europe: $489) and $2,746 for outpatients (US: $256; Europe: $6,045).

The main advantages of conducting an elective study of drug allergy are delabelling false allergic patients, who are more numerous than true allergy sufferers, and the correct diagnosis of patients with true hypersensitivity. In addition, there are other advantages, apart from economics.

Regarding beta-lactam antibiotics, patients mislabeled as allergic to beta-lactams receive alternative drugs which are generally less effective and less efficient. In this sense, Picard et al. (2013) observed additional costs over a year in 1,738 patients with suspected allergy to beta-lactams because of the use of other antibiotics for more than $15,000. Sade et al. (2003) identified a 38% increase in costs in terms of antimicrobial prescribed at discharge. Similarly, (MacLaughlin et al., 2000) showed that the mean cost of antibiotic treatments prescribed to patients labeled as allergic to beta-lactams ($26.81) was significantly higher compared to patients without allergy to these antibiotics ($16.28). In Spain, (Sastre et al., 2012) evaluated 505 hospitalized patients with a history of drug hypersensitivity and concluded that changes in treatments increased the mean cost of treatment four-fold (€273.47 per patient per day of hospitalization). Macy and Ngor (2013)) concluded that patients labeled as allergic to beta-lactams required 9.9% more days of hospitalization (0.59 days: 95% CI, 0.47–0.71) compared to controls. In addition, Chen et al. (2017) evaluated 252 patients labeled as allergic to penicillin who were hospitalized for other reasons and concluded that, after the allergy evaluation, a penicillin allergy label was removed in 228 subjects (90.5%). Another cross-sectional case-control study of hospitalized patients also concluded that antibiotic costs doubled in patients labeled with penicillin allergy (Borch et al., 2006). Moreover a penicillin allergy (Rimawi et al., 2013). In the US, an antimicrobial administration program at a tertiary hospital observed that evaluating beta-lactam allergy, removing the label in 145 of the 146 cases, resulted in an annual savings of $82,000 (Rimawi et al., 2013). Mattingly et al. (2018) observed that patients with penicillin allergy had direct drug costs during inpatient admission ranging from no difference to an additional $609 per patient respect patients without penicillin allergy. Moreover, outpatient prescription costs were estimated from $14 to $193 per patient higher for penicillin allergic patients. Moreover, in the case of allergy to beta-lactams, patients with selective hypersensitivity have different degrees of cross-reactivity, being able in these cases to check tolerance to other beta-lactam antibiotics that can be used as an alternative in certain situations. In fact, Sobrino-García et al. (2019), showed that of patients with selective hypersensitivity to amoxicillin who underwent a DPT with cephalosporins and carbapenems (82.76%), all tolerated alternative beta-lactams. Therefore, most patients could benefit from treatment with other beta-lactams. In addition to the economic consequences, treatment with non-beta-lactam antibiotics has multiple clinical implications. These include a higher incidence of infection by *Clostridium difficile*, *Vancomycin*-resistant *Enterococcus*, or methicillin-resistant *Staphylococcus aureus*, which are associated with greater number of days of hospitalization (Macy and Contreras, 2014) and readmissions. Furthermore, alternative antibiotics are often less effective than beta-lactams (vancomycin treatment for methicillin-sensitive *S. aureus* bacteremia is associated with more significant frequency of worsening of the disease (Barlam et al., 2016; McDanel et al., 2015) and more frequently leads to adverse reactions, which may contribute to the readmission of patients (Shehab et al., 2008). Suspicion of a penicillin allergy already has a direct impact on the choice of alternative antibiotics and entails the use of broader-spectrum and less effective antimicrobials, often associated with antimicrobial resistance (Shehab et al., 2008; Torres et al., 2019). In this sense, Bermingham et al. (2020) investigated the impact of being labeled as allergic to penicillin in a cohort of patients with sepsis. Their results showed that these patients frequently receive second-line antibiotics. Furthermore, they observed that the cost of alternative antibiotics in patients with suspected penicillin allergy was 2.61 times higher. Another aspect that influences the lower efficiency of antibiotic treatment in these patients is the fact that there is a delay in administering the first dose of the antibiotic (Conway et al., 2017). For its part, The United Kingdom Sepsis Trust estimates that there are about 250,000 episodes of sepsis in the United Kingdom per year. According to published data, around 20% (Mirakian et al., 2015) are associated with penicillin allergy labels that could be ruled out in 95% of cases (Shenoy et al., 2019).

Delabelling penicillin allergy is associated with greater use of penicillins and other beta-lactams. A systematic review and meta-analysis (Sacco et al., 2017) of inpatient penicillin allergy testing that included 24 studies demonstrated increase penicillin utilization (9.9–49%) after skin testing. Penicillin allergy testing in outpatient settings is also associated with significantly less health care utilization and greater use of penicillins and cephalosporins (Macy and Shu, 2017). There are regional differences in approaches for delabelling patients allergic to beta-lactams. A precise diagnosis is mainly based on skin tests and DPT tools that are time-consuming and are not without risks Torres et al., 2019. In the US, in recent years, there has been a growing interest in the development of risk stratification using a computerized clinical decision support system or a multidisciplinary antibiotic stewardship program with or without evaluation by an allergy specialist (Torres et al., 2019). It has been suggested performing DPT without previous skin tests in patients with low risk (Abrams et al., 2019;. In this sense, Li et al., 2019 concluded that penicillin allergy evaluation performing a DPT without previous STs might be feasible for adult patients with a history of type B reactions to penicillins without a history of anaphylaxis within the last ten years or a type 2, 3, or 4 (severe) hypersensitivity reaction. There is a consensus about this practice in children (Abrams et al., 2019; Stone et al., 2020).

### Beta-Lactam Studies in Children

In the case of children, it is necessary to consider that the percentage of positive results for beta-lactam antibiotics after the allergological evaluation is less than 10% (prevalence of 6% (Ibáñez and Olaguibel, 2015)), so delabelling acquires more significant importance in this population group. In this sense, Abrams et al. (2016) proposed a diagnostic protocol for children who are labeled as allergic to beta-lactams and insisted on the importance of correctly labeling the allergy to beta-lactams in the pediatric population, given its low prevalence (Seitz et al., 2011; Abrams et al., 2016; Mill et al., 2016; Roduit, 2019). Sousa-Pinto et al. (2018) identified 1,718 hospitalizations corresponding to children with suspected allergy to beta-lactams. These children had more extended days of hospitalization and a higher comorbidity rate. Hospitalization costs were also higher (€2,071 vs. €1,798), nevertheless, in this case, there was not a significant difference. Also, Au et al. (2019) estimated the costs of antibiotics used throughout their lives by patients labeled as allergic to penicillin before ten years of age compared to those who were not allergic to penicillin. Thus, they found that in the first group, the mean cost per patient was $8,171, compared to $6,278 in patients without this label.

In the study of children with suspected beta-lactam allergy (Sobrino et al.), indirect costs were higher than those of adult studies (Sobrino-García et al., 2019; Sobrino-García et al., 2020) due to the significant number of legal guardians who went to the Allergy Service and were employed, reaching 60% of cases. In the prospective study of costs of beta-lactams in children (Sobrino et al.) who went to the Allergy Service with a legal guardian who worked for hire, total costs were significantly higher (€352.70 ± 167.98) than in those whose legal guardian did not work for hire (€159.13 ± 57.29), *p* < 0.001.

### NSAIDs Studies

Concerning **NSAIDs**, Aspirin® and other drugs in this group represent one frequent cause of hypersensitivity reactions, which affect 1–3% of the population (Stevenson et al., 2001; Sánchez-Borges et al., 2010; Doña et al., 2012; Kowalski and Stevenson, 2013; Park et al., 2013; Demir et al., 2015; Kowalski and Makowska, 2015; Lipscomb et al., 2019). This percentage increases to 30% in patients with other pathologies, Szczeklik and Stevenson (2003) and there are even studies in which NSAIDs are the drugs most frequently involved in hypersensitivity reactions (Doña et al., 2011). Regarding the economic cost of hypersensitivity to NSAIDs, some studies have evaluated the costs associated with the use of alternative drugs and desensitization. In Spain, Cubero et al. (2017) confirmed that annual increase in the cost of using alternative antiplatelet agents such as clopidogrel was 1,142.12% (€218.13 vs. €17.64) and with trifusal was 662.76% (€134.56 vs. €17.64). In turn, Shaker et al. (2008) performed an economic analysis of desensitization to acetylsalicylic acid in aspirin-exacerbated respiratory disease (AERD), concluding that ambulatory desensitization is cost-effective in patients with moderate to severe AERD and that it continues to be a less expensive option for secondary cardiovascular prophylaxis.

In the only prospective study of costs of NSAID hypersensitivity (Sobrino-García et al., 2020), mean costs in patients with or without a final diagnosis of hypersensitivity were €239.53 ± 140.59 and €173.02 ± 145.71, respectively (*p* = 0.004). This difference was related to the mean number of visits necessary to reach the diagnosis: 4.23 ± 1.46 in patients diagnosed with hypersensitivity to NSAID and 3.34 ± 1.42 in whom allergy was discarded (*p* < 0.001).

In this case, being employed, or not, significantly increased the cost: €304.10 ± 172.55 in patients who worked for hire compared to €14.93 ± 62.55 in those who did not, *p* < 0.001.

Recently, the importance of correct labeling and delabelling of patients with a possible hypersensitivity to drugs (Macy, 2020; Solensky, 2020; Vyles et al., 2020) has also been highlighted in the context of SARS-CoV-2 Castells, 2020 infection. In health alert situations such as the present pandemic, it becomes more relevant to know when there is a real hypersensitivity to a drug that prevents its use, and when there is not. Thus, it is essential to study patients with a suspected reaction to a drug, carry out correct labeling and delabelling and, thus, reduce the risk for patients (Castells, 2020).

This review’s main limitations were the paucity of prospective studies about the topic and the fact that the same group performed three of them (Sobrino et al., 2020; Sobrino-García et al., 2019; Sobrino-García et al., 2020). Another limitation is that the studies’ results are not comparable due to how the studies were performed. Thus, the American studyl (Βlumenthal et al., 2018) did not include indirect costs, whereas the European studies (Sobrino et al., 2020; Sobrino-García et al., 2019; Sobrino-García et al., 2020) did. Table 1 shows a summary of the costs in the studies discussed in the review.

**TABLE 1:** Studies concerning the costs of the evaluation of drug allergy.

Study	Costs
Beta-Lactams (adults)	
Blumenthal et al. (2018)	$220 for the base case and $540 with more resources needed
Sobrino-García et al. (2019)	Mean cost of the elective evaluation of patients with suspected allergy to NSAIDs €187.49 ± 148.14, with a maximum of €789.96
Picard et al. (2013)	Additional costs over a year in 1,738 patients for more than $15,000
Sade et al. (2003)	38% increase in costs in terms of antimicrobial prescribed at discharge
MacLaughlin et al. (2000)	$26.81labeled as allergic vs. $16.28
Sousa-Pinto et al. (2020a)	Mean savings of $657 for inpatients and $2,746 for outpatients
Sousa-Pinto et al. (2020b)	Wide diversity in penicillin allergy testing practice (median values ranging from €50 for SPT to €190 DPT)
Sastre et al. (2012)	€273.47 per patient with a history of drug allergy per day of hospitalization
Rimawi et al. (2013)	Removing the label resulted in an annual savings of $82,000
Bermingham et al. (2020)	The cost of alternative antibiotics in patients with penicillin allergy labels was 2.61 times higher
Beta-Lactams (children)	
Sousa-Pinto et al. (2018)	Hospitalization costs were higher (2,071 vs. €1,798) in children with this label
Au et al. (2019)	Mean cost per patient was $8,171, compared to $6,278 in patients without this label
Sobrino-García et al. (2019)	Mean cost of the elective evaluation of patients with suspected allergy to BL: €275.27 ± €164.70, with a maximum of €746.78
NSAIDs (adults)	
Cubero et al. (2017)	Annual increase in the cost of using alternative drugs such as clopidogrel or trifusal instead of AAS: 218.13 vs. €17.64 and 134.56 vs. €17.64, respectively
Sobrino-García et al. (2020)	Mean cost of the elective evaluation of patients with suspected allergy to NSAIDs: €185.30 ± 146.77, with a maximum cost of €1,055.96

## Conclusion

An allergy evaluation in patients with suspected drug hypersensitivity is essential to establishing a correct diagnosis. Allergy testing allows for delabelling in a substantial percentage of patients with suspected drug allergy. The elective evaluation of beta-lactams and NSAID hypersensitivity is affordable and permits using more effective first-line drugs, which generally involves cost savings. In prospective European studies (Sobrino et al., 2020; Sobrino-García et al., 2019; Sobrino-García et al., 2020), the average cost of evaluating beta-lactam allergy in adults and children was €187.49 and €275.70, respectively, and the average cost of NSAID evaluation hypersensitivity was €185.30. In the prospective American study21, the average cost was $220 (€209.37) for a case and $540 (€482.45) when more resources were used. In this sense, several recent studies have shown that drug allergy evaluation is cost-saving in patients with suspected hypersensitivity to beta-lactams or NSAIDs.

Finally, we believe that all patients labeled as allergic to beta-lactams or NSAIDs should undergo an allergy study due to critical clinical and economic consequences. However, more prospective studies are needed for comprehensive cost-effectiveness analyses of this crucial issue.

## References

[B1] Abrams E. M. Atkinson A. R. Wong T. Ben-Shoshan M. (2019). The importance of delabeling β-lactam allergy in children. J. Pediatr. 204, 291–297. 10.1016/j.jpeds.2018.09.035 30322703

[B2] Abrams E. M. Wakeman A. Gerstner T. V. Warrington R. J. Singer A. G. (2016). Prevalence of beta-lactam allergy; a retrospective chart review of drug allergy assessment in a predominantly pediatric population. Allergy Asthma Clin. Immunol. 12, 59. 10.1186/s13223-016-0165-6 27956906 PMC5129666

[B3] Atanaskovic-Markovic M. Gomes E. Cernadas J. R. du Toit G. Kidon M. Kuyucu S. (2019). Diagnosis and management of drug-induced anaphylaxis in children: an EAACI position paper. Pediatr. Allergy Immunol. 30, 269–276. 10.1111/pai.13034 30734362

[B4] Au L. Y. C. Siu A. M. Yamamoto L. G. (2019). Cost and risk analysis of lifelong penicillin allergy. Clin. Pediatr. 58, 1309–1314. 10.1177/0009922819853014 31216862

[B5] Barlam T. F. Cosgrove S. E. Abbo L. M. MacDougall C. Schuetz A. N. Septimus E. J. (2016). Implementing an antibiotic stewardship program: guidelines by the infectious diseases society of America and the society for healthcare epidemiology of America. Clin. Infect. Dis. 62, e51–e77. 10.1093/cid/ciw118 27080992 PMC5006285

[B6] Bedolla-Barajas M. Delgado-Figueroa N. Pérez-Molina J. Orozco-Alatorre L. Bedolla-Pulido T. Morales-Romero J. (2018). Self-reported drug hypersensitivity amongst late adolescents in Mexico: a population based study. J Investig. Allergol. Clin. Immunol. 28, 281–282. 10.18176/jiaci.0268 30073969

[B7] Bermingham W. H. Hussain A. Bhogal R. Balaji A. Krishna M. T. (2020). The adverse impact of penicillin allergy labels on antimicrobial stewardship in sepsis and associated pharmacoeconomics-an observational cohort study (IMPALAS Study). J. Allergy Clin. Immunol. Pract. 8, 1747–1749.e4. 10.1016/j.jaip.2019.12.030 31917368

[B8] Βlumenthal K. G. Li Y. Banerji A. Yun B. J. Long A. A. Walensky R. P. (2018). The cost of penicillin allergy evaluation. J. Allergy Clin. Immunol. Pract. 6, 1019–1027. 28958738 10.1016/j.jaip.2017.08.006 PMC5862726

[B9] Borch J. E. Andersen K. E. Bindslev-Jensen C. (2006). The prevalence of suspected and challenge-verified penicillin allergy in a university hospital population. Basic Clin. Pharmacol. Toxicol. 98, 357–362. 10.1111/j.1742-7843.2006.pto_230.x 16623858

[B10] Calamelli E. Caffarelli C. Franceschini F. Saretta F. Cardinale F. Bernardini R. (2019). A practical management of children with antibiotic allergy. Acta Biomed. 90, 11–19. 10.23750/abm.v90i3-S.8157 PMC6502179 30830057

[B11] Castells M. C. (2020). Drug allergy labeling and delabeling in the coronavirus disease 2019 era. Ann. Allergy Asthma Immunol. 124, 523–525. 10.1016/j.anai.2020.04.012 32448437 PMC7242927

[B12] Chen J. R. Tarver S. A. Alvarez K. S. Tran T. Khan D. A. (2017). A proactive approach to penicillin allergy testing in hospitalized patients. J. Allergy Clin. Immunol. Pract. 5, 686–693. 10.1016/j.jaip.2016.09.045 27888034

[B13] Conway E. L. Lin K. Sellick J. A. Kurtzhalts K. Carbo J. Ott M. C. (2017). Impact of penicillin allergy on time to first dose of antimicrobial therapy and clinical outcomes. Clin. Therapeut. 39, 2276–2283. 10.1016/j.clinthera.2017.09.012 29032850

[B14] Cubero J. L. Simó Sánchez B. Millán P. Colás C. (2017). Desensibilización al ácido acetilsalicílico en pacientes con cardiopatía isquémica: ahorro de costes. Med. Intensiva 41, 446–447. 10.1016/j.medin.2016.09.012 28196671

[B15] Demir S. Olgac M. Unal D. Gelincik A. Colakoglu B. Buyukozturk S. (2015). Evaluation of hypersensitivity reactions to nonsteroidal anti-inflammatory drugs according to the latest classification. Allergy 70, 1461–1467. 10.1111/all.12689 26173603

[B16] Doña I. Blanca-López N. Cornejo-García J. A. Torres M. J. Laguna J. J. Fernández J. (2011). Characteristics of subjects experiencing hypersensitivity to non-steroidal anti-inflammatory drugs: patterns of response. Clin. Exp. Allergy 41, 86–95. 10.1111/j.1365-2222.2010.03651.x 21155908

[B17] Doña I. Blanca-López N. Torres M. J. García-Campos J. García-Núñez I. Gómez F. (2012). Drug hypersensitivity reactions: response patterns, drug involved, and temporal variations in a large series of patients. J Investig. Allergol. Clin. Immunol. 22, 363–371. 23101312

[B18] Ibáñez D. P. Olaguibel J. M. (2015). Alergológica 2015, Factores epidemiológicos, clínicos y socioeconómicos de las enfermedades alérgicas en España en 2015. Capítulo 12. Alergia infantil, 276–333.

[B19] Kowalski M. L. Makowska J. S. (2015). Seven steps to the diagnosis of NSAIDs hypersensitivity: how to apply a new classification in real practice? Allergy Asthma Immunol. Res. 7, 312–320. 10.4168/aair.2015.7.4.312 25749768 PMC4446629

[B20] Kowalski M. L. Makowska J. S. Blanca M. Bavbek S. Bochenek G. Bousquet J. (2011). Hypersensitivity to nonsteroidal anti-inflammatory drugs (NSAIDs) - classification, diagnosis and management: review of the EAACI/ENDA# and GA2LEN/HANNA*. Allergy 66, 818–829. 10.1111/j.1398-9995.2011.02557.x 21631520

[B21] Kowalski M. L. Stevenson D. D. (2013). Classification of reactions to nonsteroidal antiinflammatory drugs. Immunol. Allergy Clin. 33, 135–145. 10.1016/j.iac.2012.10.008 23639704

[B22] Li J. Shahabi-Sirjani A. Figtree M. Hoyle P. Fernando S. L. (2019). Safety of direct drug provocation testing in adults with penicillin allergy and association with health and economic benefits. Ann. Allergy Asthma Immunol. 123, 468–475. 10.1016/j.anai.2019.08.005 31419490

[B23] Lipscomb J. Wong M. Birkel M. (2019). Management of nonsteroidal anti-inflammatory drug-induced hypersensitivity reactions. U.S. Pharm. 44, 22–26.

[B24] MacLaughlin E. J. Saseen J. J. Malone D. C. (2000). Costs of beta-lactam allergies: selection and costs of antibiotics for patients with a reported beta-lactam allergy. Arch. Fam. Med. 9, 722–726. 10.1001/archfami.9.8.722 10927711

[B25] Macy E. (2020). Addressing the epidemic of antibiotic “allergy” over-diagnosis. Ann. Allergy Asthma Immunol. 124, 550–557. 10.1016/j.anai.2019.12.016 31881269

[B26] Macy E. Contreras R. (2014). Health care use and serious infection prevalence associated with penicillin “allergy” in hospitalized patients: a cohort study. J. Allergy Clin. Immunol. 133, 790–796. 10.1016/j.jaci.2013.09.021 24188976

[B27] Macy E. Ngor E. W. (2013). Safely diagnosing clinically significant penicillin allergy using only penicilloyl-poly-lysine, penicillin, and oral amoxicillin. J. Allergy Clin. Immunol. Pract. 1, 258–263. 10.1016/j.jaip.2013.02.002 24565482

[B28] Macy E. Shu Y.-H. (2017). The effect of penicillin allergy testing on future health care utilization: a matched cohort study. J. Allergy Clin. Immunol. Pract. 5, 705–710. 10.1016/j.jaip.2017.02.012 28366717

[B29] Mattingly T. J. II Fulton A. Lumish R. A. Williams A. M. C. Yoon S. Yuen M. (2018). The cost of self-reported penicillin allergy: a systematic review. J. Allergy Clin. Immunol. Pract. 6 (5), 1649–1654 .e4. 10.1016/j.jaip.2017.12.033 29355644

[B30] McDanel J. S. Perencevich E. N. Diekema D. J. Herwaldt L. A. Smith T. C. Chrischilles E. A. (2015). Comparative effectiveness of beta-lactams versus vancomycin for treatment of methicillin-SusceptibleStaphylococcus aureusBloodstream infections among 122 hospitals. Clin. Infect. Dis. 61, 361–367. 10.1093/cid/civ308 25900170

[B31] Mill C. Primeau M.-N. Medoff E. Lejtenyi C. O’Keefe A. Netchiporouk E. (2016). Assessing the diagnostic properties of a graded oral provocation challenge for the diagnosis of immediate and nonimmediate reactions to amoxicillin in children. JAMA Pediatr. 170, e160033. 10.1001/jamapediatrics.2016.0033 27043788

[B32] Mirakian R. Leech S. C. Krishna M. T. Richter A. G. Huber P. A. J. Farooque S. (2015). Management of allergy to penicillins and other beta-lactams. Clin. Exp. Allergy 45, 300–327. 10.1111/cea.12468 25623506

[B33] Modena B. White A. A. Woessner K. M. (2017). Aspirin and nonsteroidal antiinflammatory drugs hypersensitivity and management. Immunol. Allergy Clin. 37, 727–749. 10.1016/j.iac.2017.07.008 28965637

[B34] Moreno E. Laffond E. Muñoz-Bellido F. Gracia M. T. Macías E. Moreno A. (2016). Performance in real life of the European Network on Drug Allergy algorithm in immediate reactions to beta-lactam antibiotics. Allergy 71, 1787–1790. 10.1111/all.13032 27543745

[B35] Park H-S. Kowalski M. L. Sanchez-Borges M. (2013). “Hypersensitivity to aspirin and other non-steroidal antiinflammatory drugs,” in Middleton’s allergy principles and practice. 8th Edn. Philadelphia, PA: Elsevier, 1296–1309.

[B36] Park M. A. McClimon B. J. Ferguson B. Markus P. J. Odell L. Swanson A. (2011). Collaboration between allergists and pharmacists increases β-lactam antibiotic prescriptions in patients with a history of penicillin allergy. Int. Arch. Allergy Immunol. 154, 57–62. 10.1159/000319209 20664278

[B37] Picard M. Bégin P. Bouchard H. Cloutier J. Lacombe-Barrios J. (2013). Treatment of patients with a history of penicillin allergy in a large tertiary-care academic hospital. J. Allergy Clin. Immunol. Pract. 1, 252–257. 10.1016/j.jaip.2013.01.006 24565481

[B38] Rimawi R. H. Cook P. P. Gooch M. Kabchi B. Ashraf M. S. Rimawi B. H. (2013). The impact of penicillin skin testing on clinical practice and antimicrobial stewardship. J. Hosp. Med. 8, 341–345. 10.1002/jhm.2036 23553999

[B39] Roduit C. (2019). Drug allergy in children: more often suspected than real. Ther. Umsch. 75, 29–31. [in German]. 10.1024/0040-5930/a001057 31282838

[B40] Sacco K. A. Bates A. Brigham T. J. Imam J. S. Burton M. C. (2017). Clinical outcomes following inpatient penicillin allergy testing: a systematic review and meta-analysis. Allergy 72 (9), 1288–129610. 28370003 10.1111/all.13168

[B41] Sade K. Holtzer I. Levo Y. Kivity S. (2003). The economic burden of antibiotic treatment of penicillin-allergic patients in internal medicine wards of a general tertiary care hospital. Clin. Exp. Allergy 33, 501–506. 10.1046/j.1365-2222.2003.01638.x 12680867

[B42] Sagar P. S. Katelaris C. H. (2013). Utility of penicillin allergy testing in patients presenting with a history of penicillin allergy. Asia Pac. Allergy 3, 115–119. 10.5415/apallergy.2013.3.2.115 23667835 PMC3643063

[B43] Sánchez-Borges M. Caballero-Fonseca F. Capriles-Hulett A. González-Aveledo L. (2010). Hypersensitivity reactions to nonsteroidal anti-inflammatory drugs: an update. Pharmaceuticals 3, 10–18. 10.3390/ph3010010 27713240 PMC3991018

[B44] Sastre J. Manso L. Sanchez-García S. Fernández-Nieto M. (2012). Medical and economic impact of misdiagnosis of drug hypersensitivity in hospitalized patients. J. Allergy Clin. Immunol. 129, 566–567. 10.1016/j.jaci.2011.09.028 22035657

[B45] Seitz C. S. Bröcker E.-B. Trautmann A. (2011). Diagnosis of drug hypersensitivity in children and adolescents: discrepancy between physician-based assessment and results of testing. Pediatr. Allergy Immunol. 22, 405–410. 10.1111/j.1399-3038.2011.01134.x 21309856

[B46] Shaker M. Lobb A. Jenkins P. O'Rourke D. Takemoto S. K. Sheth S. (2008). An economic analysis of aspirin desensitization in aspirin-exacerbated respiratory disease. J. Allergy Clin. Immunol. 121, 81–87. 10.1016/j.jaci.2007.06.047 17716716

[B47] Shehab N. Patel P. R. Srinivasan A. Budnitz D. S. (2008). Emergency department visits for antibiotic‐associated adverse events. Clin. Infect. Dis. 47, 735–743. 10.1086/591126 18694344

[B48] Shenoy E. S. Macy E. Rowe T. Blumenthal K. G. (2019). Evaluation and management of penicillin allergy. J. Am. Med. Assoc. 321, 188–199. 10.1001/jama.2018.19283 30644987

[B49] Sobrino M. Muñoz-Bellido F. J. Macías E. Lázaro-Sastre M. de Arriba-Méndez S. Dávila I. (2020). A prospective study of costs associated with the evaluation of β-lactam allergy in children. J. Pediatr. 223, 108–113.e2. 10.1016/j.jpeds.2020.04.018 32532647

[B50] Sobrino García M. Muñoz Bellido F. J. Moreno E. Macías E. Gracia Bara M. T. Laffond E. (2019). A comprehensive prospective study of costs associated to the evaluation of beta-lactam allergy. J. Investig. Allergol. Clin. Immunol. 31 (1). Online ahead of print. 10.18176/jiaci.0457 31599727

[B51] Sobrino-García M. Muñoz-Bellido F. J. Moreno E. Macías E. Gracia-Bara M. T. Laffond E. (2020). A prospective study of costs associated to the evaluation of nonsteroidal anti-inflammatory hypersensitivity reactions. Allergy 75 (6), 1495–1497. 10.1111/all.14169 31891420

[B52] Solensky R. (2020). Doctor, I am allergic to penicillin; is this dangerous? Ann. Allergy Asthma Immunol. 124, 544–545. 10.1016/j.anai.2020.01.027 32044454

[B53] Soto-Álvarez J. (2020). Estudios de farmacoeconomía, principios y práctica. Available at: http://www.academia.cat/files/425-3261 (Accessed de febrero de 12, 2020).

[B54] Sousa-Pinto B. Araújo L. Freitas A. Delgado L. (2018). Hospitalizations in children with a penicillin allergy label: an assessment of healthcare impact. Int. Arch. Allergy Immunol. 176, 234–238. 10.1159/000488857 29788022

[B55] Sousa-Pinto B. Blumenthal K. G. Macy E. Bavbek S. Benić M. S. Alves-Correia M. (2020a). Diagnostic testing for penicillin allergy: a survey of practices and cost perceptions. Allergy 75, 436–441. 10.1111/all.13951 31230367

[B56] Sousa-Pinto B. Blumenthal K. G. Macy E. Pereira A. M. Azevedo L. F. Delgado L. (2020b). Penicillin allergy testing is cost-saving: an economic evaluation study. Clin. Infect. Dis., ciaa194. 10.1093/cid/ciaa194 PMC7958749 32107530

[B57] Stevenson D. D. Sanchez-Borge M. Szczeklik A. (2001). Classification of allergic and pseudoallergic reactions to drugs that inhibit cyclooxygenase enzymes. Ann. Allergy Asthma Immunol. 87, 177–180. 10.1016/s1081-1206(10)62221-1 11570612

[B58] Stone C. A. Trubiano J. Coleman D. T. Rukasin C. R. F. Phillips E. J. (2020). The challenge of de‐labeling penicillin allergy. Allergy 75, 273–288. 10.1111/all.13848 31049971 PMC6824919

[B59] Szczeklik A. Stevenson D. D. (2003). Aspirin-induced asthma: advances in pathogenesis, diagnosis, and management. J. Allergy Clin. Immunol. 111, 913–921. 10.1067/mai.2003.1487 12743549

[B60] Torres M. J. Adkinson N. F. Jr. Caubet J.-C. Khan D. A. Kidon M. I. Mendelson L. (2019). Controversies in drug allergy: beta-lactam hypersensitivity testing. J. Allergy Clin. Immunol. Pract. 7 (1), 40–45. 10.1016/j.jaip.2018.07.051 30245291

[B61] Torres M. J. Blanca M. Fernández J. Romano A. Weck A. Aberer W. (2003). Diagnosis of immediate allergic reactions to beta-lactam antibiotics. Allergy 58, 961–972. 10.1034/j.1398-9995.2003.00280.x 14510712

[B62] Torres M. J. Moreno E. Fernandez-Santamaría R. Doña I. Fernández T. D. (2019). Diagnostic approximation to delabeling beta-lactam allergic patients. Curr. Treat. Options Allergy 6, 56–70. 10.1007/s40521-019-0202-z

[B63] Vyles D. Antoon J. W. Norton A. Stone C. A. Trubiano J. Radowicz A. (2020). Children with reported penicillin allergy. Ann. Allergy Asthma Immunol. 124, 558–565. 10.1016/j.anai.2020.03.012 32224207 PMC7255916

[B64] Wöhrl S. (2018). NSAID hypersensitivity - recommendations for diagnostic work up and patient management. Allergo J. Int. 27, 114–121. 10.1007/s40629-018-0064-0 29974031 PMC6004000

[B65] World Bank (2019). GPD per capita (current US dollar). World Bank national accounts data, and OECD National Accounts data files. Available at: https://data.worldbank.org/indicator/ny.gdp.pcap.cd (Accessed November 17, 2019).

